# Characterization and Genomic Analysis of Marinobacter Phage vB_MalS-PS3, Representing a New Lambda-Like Temperate Siphoviral Genus Infecting Algae-Associated Bacteria

**DOI:** 10.3389/fmicb.2021.726074

**Published:** 2021-08-25

**Authors:** Yundan Liu, Kaiyang Zheng, Baohong Liu, Yantao Liang, Siyuan You, Wenjing Zhang, Xinran Zhang, Yaqi Jie, Hongbing Shao, Yong Jiang, Cui Guo, Hui He, Hualong Wang, Yeong Yik Sung, Wen Jye Mok, Li Lian Wong, Andrew McMinn, Min Wang

**Affiliations:** ^1^College of Marine Life Sciences, Frontiers Science Center for Deep Ocean Multispheres and Earth System, Institute of Evolution and Marine Biodiversity, Ocean University of China, Qingdao, China; ^2^Department of Hospital Infection Management, Qilu Hospital, Shandong University, Qingdao, China; ^3^UMT-OUC Joint Centre for Marine Studies, Qingdao, China; ^4^College of Letters and Science, University of Wisconsin–Madison, Madison, WI, United States; ^5^Institute of Marine Biotechnology, Universiti Malaysia Terengganu, Kuala Terengganu, Malaysia; ^6^Institute for Marine and Antarctic Studies, University of Tasmania, Hobart, TAS, Australia; ^7^The Affiliated Hospital of Qingdao University, Qingdao, China

**Keywords:** bacteriophage, Marinobacter, hydrocarbon biodegradation, genome and comparative genome analysis, phylogenetic analysis, algal-associated bacteria

## Abstract

Marinobacter is the abundant and important algal-associated and hydrocarbon biodegradation bacteria in the ocean. However, little knowledge about their phages has been reported. Here, a novel siphovirus, vB_MalS-PS3, infecting *Marinobacter algicola* DG893(T), was isolated from the surface waters of the western Pacific Ocean. Transmission electron microscopy (TEM) indicated that vB_MalS-PS3 has the morphology of siphoviruses. VB_MalS-PS3 was stable from −20 to 55°C, and with the latent and rise periods of about 80 and 10 min, respectively. The genome sequence of VB_MalS-PS3 contains a linear, double-strand 42,168-bp DNA molecule with a G + C content of 56.23% and 54 putative open reading frames (ORFs). Nineteen conserved domains were predicted by BLASTp in NCBI. We found that vB_MalS-PS3 represent an understudied viral group with only one known isolate. The phylogenetic tree based on the amino acid sequences of whole genomes revealed that vB_MalS-PS3 has a distant evolutionary relationship with other siphoviruses, and can be grouped into a novel viral genus cluster with six uncultured assembled viral genomes from metagenomics, named here as *Marinovirus.* This study of the Marinobacter phage vB_MalS-PS3 genome enriched the genetic database of marine bacteriophages, in addition, will provide useful information for further research on the interaction between Marinobacter phages and their hosts, and their relationship with algal blooms and hydrocarbon biodegradation in the ocean.

## Introduction

The *Marinobacter* genus is within the class Gammaproteobacteria, order *Oceanospirillales* and includes Gram-negative, aerobic, motile, halotolerant or halophilic, rod-shaped bacteria (Gauthier et al., 1992), and well known for algal-associated bacteria and its capacity of hydrocarbon degradation, particularly using aromatic and aliphatic hydrocarbons as its sole carbon source (Martin et al., 2003; Holtzapple and Schmidt-Dannert, 2007; Kim et al., 2017). *Marinobacter* genus is one of the important algal-associated bacterial groups in marine environments (Gonzalez et al., 2000; Buchan et al., 2014). *Marinobacter algicola* DG893(T) was isolated from paralytic shellfish toxin-producing dinoflagellates (Green et al., 2006), and had been proposed to generate photoactive siderophores that may offer a bioavailable form of iron to commensally associated phytoplankton, affecting algal growth and bloom dynamics (Yarimizu et al., 2018). In addition, petroleum pollution has become a serious environmental problem in the sea, along with the occasional and various release routes such as oil mining, accidental oil spills, shipping transportation, and industrial activities (Cui et al., 2008). Although many marine environments are seriously affected by hydrocarbon pollution, many microbes can utilize hydrocarbon as carbon sources and mediate the influences from hydrocarbon pollution (Antunes et al., 2007; Zenati et al., 2018). Most isolated *Marinobacter* strains from hydrocarbon-contaminated environments have been found to be able to efficiently degrade hydrocarbon and petroleum compounds (Martinez et al., 2003; Kim et al., 2006). Currently, the genus *Marinobacter* contains 81 species, and *Marinobacter aquaeolei* and its heterotypic synonym *Pseudomonas nautica* are the main hydrocarbon-degrading species (Hedlund et al., 2001; Gorshkova et al., 2003; Gu et al., 2007). *Marinobacter squalenivorans* and *Marinobacter alkaliphilus* also have the capacity to degrade hydrocarbon compounds (Shieh et al., 2003; Singer et al., 2011). These *Marinobacter* species have developed complex and diverse metabolic capacities to adapt to polluted and sometimes hypersaline environments. They can degrade hexadecane, fluoranthene, pristine, and octadecane and even metabolize the herbicide 1, 3-diphenylurea (Takai et al., 2005). The degradation of these hydrocarbon compounds can result in wax ester accumulation in the cytoplasm and influence their fatty acid composition (Hickford et al., 2004). The degradation products, however, can play key roles in marine systems, such as promoting the utilization of hydrocarbons and facilitating the absorption of nutrients (Gardner et al., 2004). This genus is potentially involved in complex ecological networks across trophic levels and could be ecologically and evolutionarily influenced by their bacteriophages (Amarillas et al., 2017).

Bacteriophages, i.e., bacterial viruses, are the most abundant and diverse biological entities on the Earth, with a total abundance of approximately 10^30^–10^32^ (Hammerl et al., 2016). The genomic and metagenomic analysis illustrated the tremendous genetic diversity and the huge unknown gene pool of bacteriophages (Li et al., 2016; Gong et al., 2018; Liang et al., 2019). By infecting and lysing their host cells, phages can control and regulate the abundance and structure of microbial communities and play an important role in global biogeochemical cycles (Pourcel et al., 2017). Viral lysing shunts microbial biomass into dissolved organic matter (DOM), mediating the recycling of macro- and micro-elements such as C, P, N, S, and Fe (De Leeuw et al., 2017). In addition, the expression of phage-encoded auxiliary metabolic genes (AMGs), such as *dsrC* and *amoC* genes, can manipulate the metabolic reprogramming of infected cells and enhance phage fitness (Zimmerman et al., 2020). However, more than 90% of the viral populations remain unknown (Gregory et al., 2019).

Despite the ecological importance and environmental remediation significance of the *Marinobacter* genus, our knowledge about their phages and potential interactions with host cells is still rare. To date, only two Marinobacter phages have been reported, including PS6 and AS1, which were isolated from the Bohai Sea of China and the Arabian Sea, respectively (as of May 2021; Zhu et al., 2018; Aparna et al., 2019). Both phages belong to the *Siphoviridae* family within the Caudovirales order, which typically has a long, flexible, and non-retractable tail (Amgarten et al., 2017; Ramphul et al., 2017). Compared with the booming metagenomic research, there are fewer studies describing the isolation and research of new marine bacteriophages. Therefore, there is a lack of culturable counterparts in the metagenomic viral sequences, which hinders the understanding of the ecological function and biological characteristics of marine viruses (Yang et al., 2021). In addition, isolation and genomic analysis of more Marinobacter phages could improve our understanding of their evolution, interaction with host cells, and potential roles in phytoplankton blooms and hydrocarbon-contaminated environments.

In this study, a new Marinobacter phage vB_MalS-PS3 was isolated from the surface water of the western Pacific Ocean and enriched the genetic database of Marinobacter bacteriophages. Comparative genomes of Marinobacter phages vB_MalS-PS3, AS1, and PS6 were also examined. Due to its novelty in the evolutionary relationship, vB_MalS-PS3 can be grouped into a novel viral cluster (VC) with six uncultured phage contigs from metagenomics, in addition, will provide useful information for further research on the interaction between *Marinobacter* and their phages. The genome sequence information reported here will provide an important basis for further study of the adaptive evolution and ecological role of Marinobacter phage and their hosts in the sea.

## Materials and Methods

### Phage Isolation, Purification, and Cross Infectivity Test

The 1 L sample water was collected from the western Pacific Ocean (10.6°N, 143.6167°E), during the spring of 2017, and filtered through 0.22 μm pore-size membranes (Millipore) and checked for the presence of phages by using the double-agar layer method (0.5% low-melt top agar) to obtain the plaques. Each plaque was cored, plaque-purified three times and resuspended in 500 μL of sterile SM buffer [100 mM NaCl, 81.2 mM MgSO_4_. 7H_2_O, 50 mM Tris–HCl (pH 7.5), 0.01% gelatin]. Purified bacteriophage was stored in SM buffer at 4°C for a few months for further processing (Liu et al., 2019). The host bacterial strain *M. algicola* DG893(T) was incubated in LB liquid medium at 28°C.

In order to establish the infection efficacy of each phage, host range tests were evaluated on a range of *Marinobacter* species by using a cross infectivity test (Table 1). 200 μl of bacteriophage suspension (10^8^ PFU) was mixed with 200 μl of an overnight bacterial culture undergoing exponential growth (OD = 600) and with 4.5 ml of top agar (cooled to 45°C) and poured onto the LB agar, SM buffer alone was used as a blank control (Kajsík et al., 2019). After overnight cultivation at 28°C the plaques were counted by the double-layer agar method. The occurrence of plaques forming in the plate indicated the presence of phages.

**TABLE 1:** Bacterial strains used in this study to determine phage host range.

**Bacterial species**	**Source**	**Infectivity**
*Marinobacter algicola* DG893(T)	a	+
*Marinobacter salarius* R9SW1	a	−
*Marinobacter hydrocarbonoclasticus* SP17^T^	a	−
*Marinobacter aquaeoli* VT8^T^	a	−
*M. algicola* DG893(T)		
*Marinobacter* sp. CAB DSM11874	a	−
*Marinobacter pelagius* HS225^T^	a	−
*Marinobacter salsuginis* DSM18347^T^	a	−
*Marinobacter alkaliphilus’* JCM1229	a	−
*Marinobacter squalenivorans* DSMZ15125	a	−

*a: strains were purchased from the Marine Culture Collection of China (MCCC, China). T: type strain.*

### Transmission Electron Microscopy

The morphology of the phage was examined by transmission electron microscopy (TEM). The purified phage particles were negatively stained with phosphotungstic acid (1% w/v, pH 7.0; Zhang et al., 2020). Stained particles were observed using a JEOL Model JEM-1200EX TEM at 100 kV. Phage dimensions were estimated based on the electron micrographs (Gao et al., 2017).

### One-Step Growth Curve Assay and Biological Feature Assays

A modified soft-agar overlay method was used to test the one-step growth curve assay. The phage vB_MalS-PS3 was mixed with the host bacterium in the exponential growth phase (OD600) at a multiplicity of infection (MOI) of 0.1 and incubated at 37°C. To allow adsorption of the phages to the host bacteria the suspensions were incubated for 15 min before further centrifugation, then the infected culture was centrifuged at 8,000 × *g* for 1 min to remove the unabsorbed phages. Five ml of fresh LB liquid medium was used to suspend the cells. Samples were taken at 5-min intervals (up to 30 min) and 10-min intervals (up to 90 min) and the phage titer was then measured by plaque assays (Kwiatek et al., 2017).

To investigate phage stability at different temperatures, SM buffer was used to dilute phage vB_MalS-PS3. The mixtures were then incubated at −20, 4, 25, 45, 55, 65, 75, and 85°C for 2 h; the double-layer agar method was used to calculate the survival rate of each treated sample. To determine the phage capsid stability, the vB_MalS-PS3 phage was incubated over a pH range of 2–13 with 1 M NaOH or 1 M HCl for 2 h at adjusted optimum temperature; the double-layer agar method was used to evaluate the phage survival rate (Wang et al., 2017).

### Extraction of Phage DNA, Sequencing, and Bioinformatic and Phylogenetic Analysis

Bacteriophage genomic DNA extraction was performed by Virus DNA Kit (OMEGA), utilizing DNase and RNase. Nucleic acids were detected by electrophoresis (Pires et al., 2011). Purified phage genomic DNA was sequenced using Illumina Miseq 2 × 300 paired-end sequence by the Novogene Company (Tianjin), the raw data were then filtered to get clean data and assembled using SOAP *de novo* v2.04 (Beilstein and Dreiseikelmann, 2006).

Open reading frames (ORFs) were predicted using a combination of Glimmer,^1^ RAST,^2^ and GeneMarkS^3^ (Schneiker et al., 2006). The initiation codon and terminate codon of each ORF were checked manually to avoid breakdown for long protein sequence. The sequences of ORFs were subjected to the BLASTp against by the non-redundant (nr) protein database of the GenBank,^4^ with *E*-values < 1E-5. Pfam^5^ with default parameters also were used to predict the function of the proteins (Mistry et al., 2020). tRNAs were predicted by the tRNAscan-SE program.^6^ The comparative genome figure was produced using Easyfig (Meng et al., 2017). Protein domains were predicted and analyzed using InterPro^7^ and CDD.^8^ Genome mapping was performed using CLC Main Workbench 20. The bacteriophage proteomic tree and genome alignments were conducted with Viptree^9^ (Nishimura et al., 2017). Viral genome sequences and taxonomic information of viruses and their hosts are based on the Virus-Host DB using tBLASTx (Mihara et al., 2016). Selected 1,265 viral genomes as reference sequences to build a phylogenetic tree, and 33 closely related genome sequences of vB_MalS-PS3 were selected to draw a rectangular proteomic tree. The heatmap of the shared genes between 32 closely related genome sequences of vB_MalS-PS3 was plotted using the ‘‘pheatmap’’ R package. The whole-genome phylogenetic tree based on amino acid sequences was constructed using the Virus Classification and Tree Building Online Resource (VICTOR^10^) and visualized with iTol (v.5; Meier-Kolthoff and Göker, 2017; Letunic and Bork, 2019). All pairwise comparisons of the nucleotide sequences were conducted using the genome-blast distance phylogeny (GBDP) method under settings recommended for prokaryotic viruses (Meier-Kolthoff et al., 2013; Meier-Kolthoff and Göker, 2017). Taxon boundaries at the species, genus and family level were estimated with the OPTSIL program, the recommended clustering thresholds and an *F* value (fraction of links required for cluster fusion) of 0.5 (Göker et al., 2009; Meier-Kolthoff et al., 2014; Meier-Kolthoff and Göker, 2017). The average nucleotide identity (ANI) was calculated using OrthANI software and JSpeciesWS^11^ to establish ANI phylogenetic trees (Lee et al., 2016; Richter et al., 2016).

### Phage Homologs in Environmental Viral Sequences and Network Analysis

In order to know the accurate taxonomy information and search for homologs of vB_MalS-PS3, each coding sequence was queried against Integrated Microbial Genomes/Virus (IMG/VR) and Global Ocean Viromes 2.0 (GOV 2.0) database using all-versus-all BLASTp, with an *E* value cutoff of 10^–5^ (Paez-Espino et al., 2016; Gregory et al., 2019). VCs were identified using ClusterONE with default parameters which are defined in the vConTACT 2.0 (Nepusz et al., 2012; Jang et al., 2019). Network visualization and modular analysis were made by Gephi version 0.9.2 (Xue et al., 2018).

### Genome Sequence Accession Number

The annotation results and related information were uploaded to GenBank to obtain the accession number. The complete genome sequence of bacteriophage vB_MalS-PS3 is available in the GenBank database under accession number MF959999.

## Results and Discussion

### Biological Features and Host Range of Phage vB_MalS-PS3

A novel Marionbacter phage vB_MalS-PS3 was isolated from the western Pacific Ocean, serving *M. algicola* DG893(T) as its host. Phytoplankton blooms can cause acute effects on marine ecosystems, one hypothesis is that bacteria producing photoactive iron carriers can provide a bioavailable form of iron for symbiotic phytoplankton, which in turn will affect the growth and bloom dynamics of algae (Yarimizu et al., 2018). *M. algicola* DG893(T) is a phytoplankton-associated bacterium that produces the photoactive siderophore vibrioferrin (Romano et al., 2013). Therefore, vB_MalS-PS3, the phage hosted by algae-associated bacteria, might influence the population dynamics of harmful algal blooms through influencing their host cells.

**FIGURE 1:**
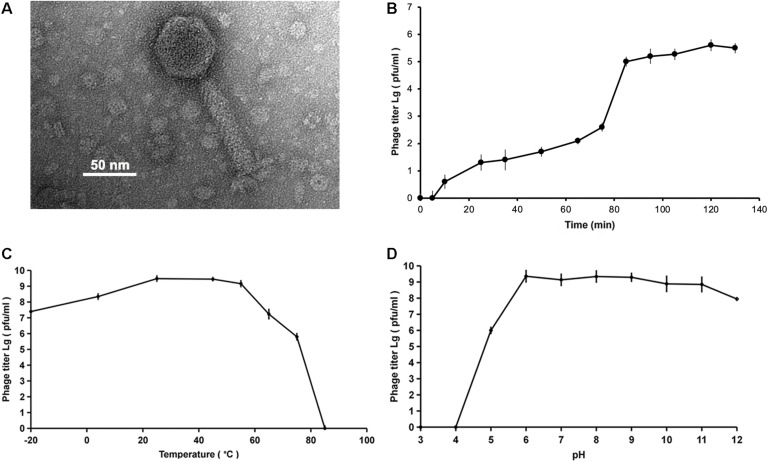
**(A)** Transmission electron microscopy (TEM) of bacteriophage vB_MalS-PS3. The scale bar is 50 nm. **(B)** One-step growth curve of vB_MalS-PS3. **(C)** The curve of thermal stability of vB_MalS-PS3. **(D)** The curve of capsid stability of vB_MalS-PS3. The *Y*-axis of the figure is a log scale.

The bacteriophages were examined by TEM and classified on the basis of their morphological features to the order Caudovirales, tailed phages. Phage vB_MalS-PS3 has an icosahedron head (∼50 nm in diameter) and a long, non-contractile tail (∼100 nm), it was further classified into the *Siphoviridae* family based on siphoviral morphology (Figure 1A). The head is a protein capsid like icosahedron and the tail contains the tail tube, tail plate, and tail filament.

The one-step growth curve showed that the latent period is approximately 80 min and the rise periods is about 10 min (Figure 1B). When the ambient temperature was between −20 and 55°C, the titer of vB_MalS-PS3 remained at a high level (Figure 1C). Optimum temperature of phage infection is 25°C. The phage titer dropped sharply when temperature up to 60°C and the maximum temperature that vB_MalS-PS3 maintains infectious activity is around 65°C. For capsid stability, no phage titer was detected when the pH was less than or equal to three. From pH 6 to 11, there is no significant loss of phage titer (Figure 1D). A significant decline of titer occurred when the pH was equal to or greater than 12.

From the result of cross-infection using double-layer medium, phage vB_MalS-PS3 had a narrow host range, which could not infect the other eight species tested in the *Marinobacter* genus expect *M. algicola* DG893(T) (Table 1).

### Lambda-Like Genomic Elements Encoded by vB_MalS-PS3 With Two AMGs

The genome of vB_MalS-PS3 consists of a linear, double-stranded 42,168-bp DNA molecule with a GC content of 56.23% and no tRNA genes. The coding ratio of the phage genome is 89.27%.

**FIGURE 2:**
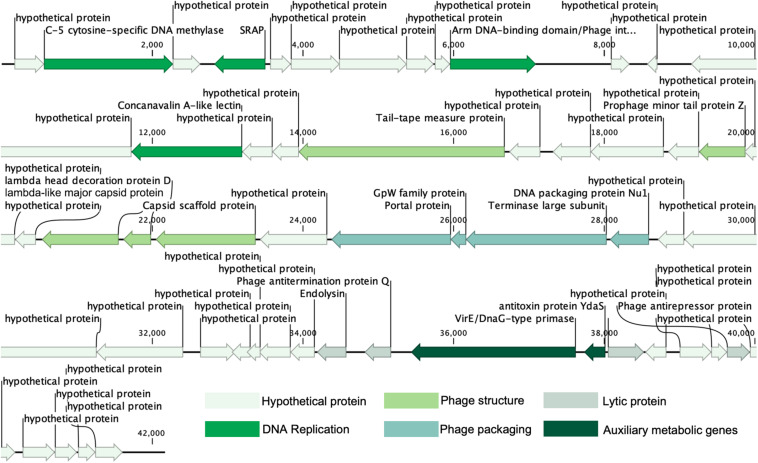
Genome map and functional annotation of the predicted proteins of vB_MalS-PS3.

The results of the prediction showed that the genome contains 54 ORFs (Figure 2 and Table 2), among them, in the positive strand, there were 20 ORFs, the other 34 ORFs were present in the negative strand. Most of the ORFs (45) are initiated by codon ATG, and nine are initiated by alternative codon GTG. From the result of ORFs annotations, it was found that 29 ORFs have high similarity with other published viral genes. Nineteen ORFs possess known functions. Other ORFs (*n* = 35) are ORFans for missing homologous proteins in current public databases.

**TABLE 2:** The conserved domains detected of vB_MalS-PS3.

**ORF**	**Start**	**Stop**	**Strand**	**Function**	**Match phage**	**Conserved domains**
2	561	2,273	+	C-5cytosine-specific DNA methylase	Pseudomonas phage LKA5	PF00145
4	3,501	2,824	−	SOS response associated peptidase (SRAP)	Stenotrophomonas phage S1	PF02586
10	5,953	7,092	+	ArmDNA-binding domain/Phage integrase	Escherichia virus Lambda_4A7	PF09003/PF00589
14	13,192	9,153	−	Concanavalin A-like lectin	Uncultured Caudovirales phage	PF13385
17	16,678	13,943	−	Tail-tape measure protein (TMP)	Dinoroseobacter phage vB_DshS-R5C	PF10145
22	19,875	19,258	−	Prophage minor tail protein Z	Prokaryotic dsDNA virus sp.	PF06763
25	21,553	20,534	−	Lambda-like major capsid protein	Escherichia phage Lambda_ev017	PF03864
26	21,983	21,615	−	Lambda head decoration protein D	Alteromonas phage P24	PF02924
27	23,371	22,064	−	Capsid scaffold protein	Escherichia phage Lambda	PF01343
29	25,964	24,378	−	Portal protein	Escherichia phage Lambda	PF05136
30	26,167	25,961	−	GpW family protein	Escherichia phage Lambda	PF02831
31	28,032	26,164	−	Terminase large subunit	Escherichia phage Lambda	PF05876
32	28,594	28,076	−	Phage DNA packaging protein Nu1	Escherichia phage Lambda	PF07471
41	34,576	34,187	−	Endolysin	Escherichia phage vB_EcoM_PHB13	PF16083
42	35,170	34,820	−	Phage antitermination protein Q	Escherichia phage Lambda_ev087	PF06530
43	37,622	35,436	−	Virulence-associated protein E(VirE)/DnaG-type primase	Escherichia phage vB_EcoM_ECOO78	PF05272/PF13362
44	38,015	37,734	−	Antitoxin protein YdaS	Escherichia phage phi467	PF15943
45	38,048	38,527	+	Phage antirepressor protein	Enterobacteria phage phi80	PF03374
49	39,629	39,934	+	Phage antirepressor protein	Prokaryotic dsDNA virus sp.	PF03374

Nineteen conserved protein domains were detected (Table 2). Three viral genetic functional modules based on these protein domains were classified, including DNA replication and nucleotide metabolism, phage structure and additional functions (phage packaging, lysis).

ORF10 is the integrase of vB_MalS-PS3, indicating its temperate propagation property in natural habitats. It can catalyze site-specific integration and excision of temperate bacteriophages and other mobile genetic elements from the bacterial host chromosome and shows similarity to Escherichia virus Lambda_4A7 (Tay et al., 2010). Two functional domains were detected in this ORF, including the Arm DNA-binding domain (PF09003) and the Phage integrase family (PF00589). PF09003 and PF00589 locate at the beginning of ORF10 (amino acids sites: from 1 to 63) and another segment (from 188 to 357), respectively. PF09003 is a part of lambda-like integrase, which is responsible for high-affinity binding to each of the five DNA arm-type sites and is also a context-sensitive modulator of DNA cleavage (Wojciak et al., 2002). These two domains are prevalent in *Siphoviridae* based on the information provided by Pfam, especially for those temperate siphoviruses. Similar integration and recombination progress with Escherichia phage Lambda might be applied by phage vB_MalS-PS3. ORF2, coding the enzyme of C-5 cytosine-specific DNA methylase, modifies the phage DNA to avoid the degradation or restriction of enzymes from the host cell, or methylation marks the phage DNA to protect it from its own nucleases which degrade the host DNA during the early stages of infection (Aravind et al., 2013). From current information of this protein in Pfam (PF00145), most viral-encoded C-5 cytosine-specific DNA methylase is from *Siphoviridae* (*n* = 264), occupying 44.5% number of related sequences in viruses. This indicates this enzyme might be much more prevalent in *Siphoviridae* than other viruses. ORF2 shows homology to Pseudomonas phage LKA5, which might play an important role in the lysogenic life cycle of vB_MalS-PS3. ORF4 coding proteins, which have a similarity to known virus proteins in the database, contained a conservative field, which is related to the peptide enzyme (SRAP) in the SOS response. It can thus be concluded that ORF4-encoded proteins may be associated with DNA repair. Abasic (AP) sites are one of the most common DNA lesions that block replicative polymerases. SRAP proteins shield the AP site from endonucleases and error-prone polymerases (Mohni et al., 2019). Thus, the expression of SRAP encoded by vB_MalS-PS3 might protect the replication of viral genomes from occasional interruption inside cells.

Two protein domains were included in ORF43, including Toprim domain (PF13362; from 203 to 307) and Virulence-associated protein E (PF05272; from 429 to 619). PF13362 is a DnaG-type primase (Aravind and Landsman, 1998), while PF05272 is a typical AMG. ORF43 is homologous with Escherichia phage vB_EcoM_ECOO78 and contains a P-loop motif. Protein with the domain of PF05272 can aggravate the pathogenicity of some pathogenic bacteria (Ji et al., 2016) so that ORF43 might promote interspecific or intraspecific competition ability for those vB_MalS-PS3-infected *Marinobacter*. ORF 44 encodes antitoxin protein YdaS (PF15943) and shows homologous with Escherichia phage phi467. YdaS is a putative bacterial antitoxin that can neutralize the toxin YdaT, while YdaS is an AMG. This entry also includes some bacteriophage proteins, such as cro from Pseudomonas phage D3 and gene 30 protein from Enterobacteria phage phi80 (Yamaguchi and Inouye, 2011).

ORF29, ORF 30, ORF31, and ORF32 encode four packaging-associated genes, including portal protein, GpW family protein, terminase large subunit and Phage DNA packaging protein Nu1, respectively. All ORFs show homology to the Escherichia phage Lambda, which contains Lambda family phage portal protein (PF05136), gpW (PF02831), Terminase_GpA domain (PF05876), and Phage_Nu1domain (PF07471), respectively. Despite the Lambda-like terminase homolog was found in the genome of vB_MalS-PS3, it is still unknown whether the Lambda-like-terminase-mediated process could participate in the DNA packaging of vB_MalS-PS3. Hence, the functions of Lambda-like terminase in DNA packaging of vB_MalS-PS3 is still unclear. We hypothesized that it might introduce staggered nick to generate the cohesive ends (similar to cosN) from other specific site recognition to implement the function of DNA shearing. This also confirmed the potential highly hybridized genotype of PS3 to some extent. Different viral gene possesses a divergent origin, suggesting the special phylogenic status and evolutionary history of PS3. As a series of lambda-homologous proteins found in the genome of PS3, which might be the gene signature of viral evolutionary history of PS3, we speculate that this phage might be originated from *Lambdavirus* in the past, but acquired or loss some other genes. Hence, the PS3 was not clustered with *Lambdavirus* in the phylogenetic tree.

Among the proteins directly involved in phage structure, ORF25 is a Lambada-like major capsid protein with Phage major capsid protein E domain (PF03864). ORF 22 is a Prophage minor tail protein Z (PF06763), and this family consists of several prophage minor tail protein Z like sequences from *Escherichia coli*, Salmonella typhimurium and Lambda-like bacteriophages. ORF17 encodes the tail-tape measure protein (TMP) and shows homology to Dinoroseobacter phage vB_DshS-R5C. Forty-four isolated viruses with homologs of ORF17 were reported in GenBank, and the genome size of all related phages infecting Proteobacteria was higher than 70,000-bp. To the best of our knowledge, vB_MalS-PS3 is the only small-genome Proteobacteria phages encoding vB_DshS-R5C-like TMP. ORF27 is a capsid scaffold protein with Peptidase family S49 domain (PF01343), which is homologous with Escherichia phage Lambda and other several lambda-like phages. Several genes associated with genome replication that are commonly found in phages, such as helicase, DNA-binding protein and ligase, are missing from the genome of vB_MalS-PS3 based on current annotation information. Potentially, other unknown viral replication genes may be encoded by the vB_MalS-PS3 genome, or its replication process depends on the host enzyme system, which is unclear currently.

From the view of the genomic signature of vB_MalS-PS3, it is a Lambda-like temperate siphoviruses with a hybrid viral genome. Both replication and structure functional modules contained Lambda-like genetic elements. The genes of phage vB_MalS-PS3 are from diverse siphoviral lineages, such as *Lambdavirus*, *Nanhaivirus*, and *Chivirus*.

### Phage vB_MalS-PS3 Represented a New Siphoviral Genus

**FIGURE 3:**
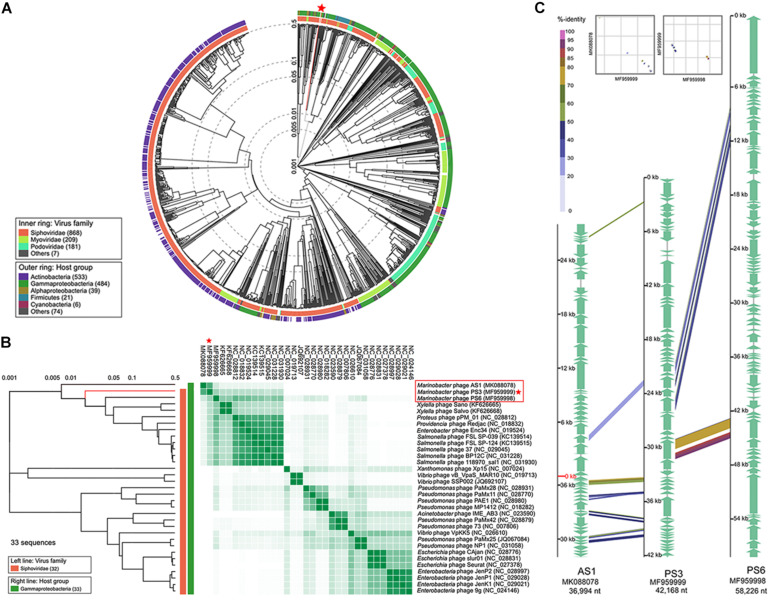
Phylogenetic and comparative genomic analysis of vB_MalS-PS3. **(A)** Viral phylogenetic tree of whole phage vB_MalS-PS3 genomes represented in the circular view. **(B)** Viral phylogenetic tree represented in the rectangular view with 32 closely related genome sequences of vB_MalS-PS3, and heatmap showing the percentage of shared genes among 33 genome sequences. The ratio of shared genes was based on all-to-all BLASTP analysis (*E*-value < 1E-10). **(C)** Genomic organization of the Marinobacter bacteriophage vB_MalS-PS3 compared to Marinobacter bacteriophage AS1 and Marinobacter bacteriophage PS6.

To identify the exact taxonomic position of the phage vB_MalS-PS3, the phylogenetic tree based on the viral proteome of whole genomes was established by Viptree (Figure 3A), and the 32 closest phages in the proteome tree were selected together with Marinobacter phage vB_MalS-PS3 to establish a rectangular tree for subsequent comparison and analysis (Figure 3B). The results of phylogenetic analysis and gene-shared heatmap revealed that vB_MalS-PS3 represents a separate cluster and is far from the other isolated phages. To confirm this finding, we chose two phages complete genome sequences (Marinobacter phage AS1 and Marinobacter phage PS3), carried out the comparative genomic analysis with vB_MalS-PS3 (Figure 3C). Similar to the results of the phylogenetic tree analysis, vB_MalS-PS3 showed low similarity in either Marinobacter phage AS1 or Marinobacter phage PS6. The results indicated the novelty of vB_MalS-PS3 and had a relatively distant evolutionary relationship which represents a novel VC.

**FIGURE 4:**
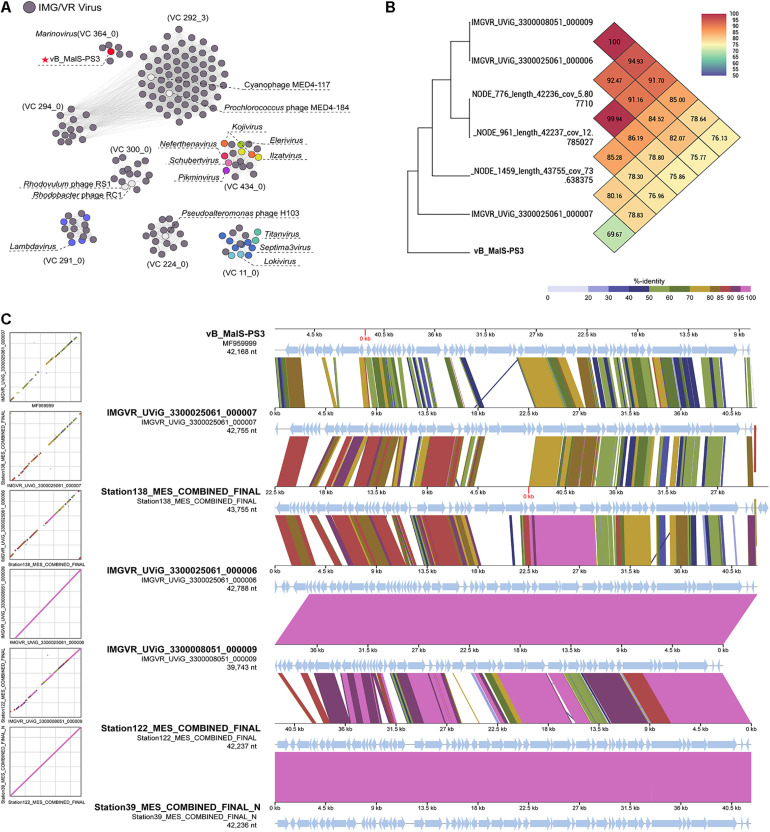
**(A)** Gene-content-based viral network of vB_MalS-PS3, *Siphoviridae* virus from NCBI RefSeq database, and related environmental viral sequences from Integrated Microbial Genomes/Virus (IMG/VR). The nodes represent the viral genomic sequences. The edges represent the similarity score between genomes based on shared gene content. The gray circles indicate the related environmental viral sequences from IMG/VR. In each viral cluster (VC), viral genomes that belong to different genus clusters classified and ratified by the International Committee on Taxonomy of Viruses (ICTV) are indicated by different colors. VCs generated using vConTACT2, the network was visualized using Gephi version 0.9.2. **(B)** Genome comparison between the vB_MalS-PS3 and the environmental viral sequences in IMG/VR. **(C)** Heat map based on OrthoANI values calculated using OAT software.

Although vB_MalS-PS3 exhibits no significant genomic synteny with other known phage isolates, high similarity with some metagenomic assembled genome virals (MAGs) from IMG/VR and GOV 2.0 database using all-versus-all BLASTp. Therefore, six MAGs with high similarity (shared genes > 50%) to vB_MalS-PS3 were retrieved from IMG/VR datasets. Then, 168 viruses (28 RefSeq from NCBI, 139 IMG/VR and GOV 2.0 metagenomic viral sequences and vB_MalS-PS3) were classified into different VCs using the vConTACT 2.0 (Figure 4A). Six phages and vB_MalS-PS3 were grouped as a VC (VC 364_0) from the result of genome-content-based analysis (Supplementary Table 1). In further comparative genomic analysis, all six phages clustered with vB_MalS-PS3 revealed considerably conserved in whole-genome synteny. Most of the homologous ORFs throughout these phage genomes located in the packaging module (portal protein, terminase large subunit and phage DNA packaging) and structural module (TMP, major capsid protein, head decoration protein D and capsid scaffold protein; Figure 4C).

**FIGURE 5:**
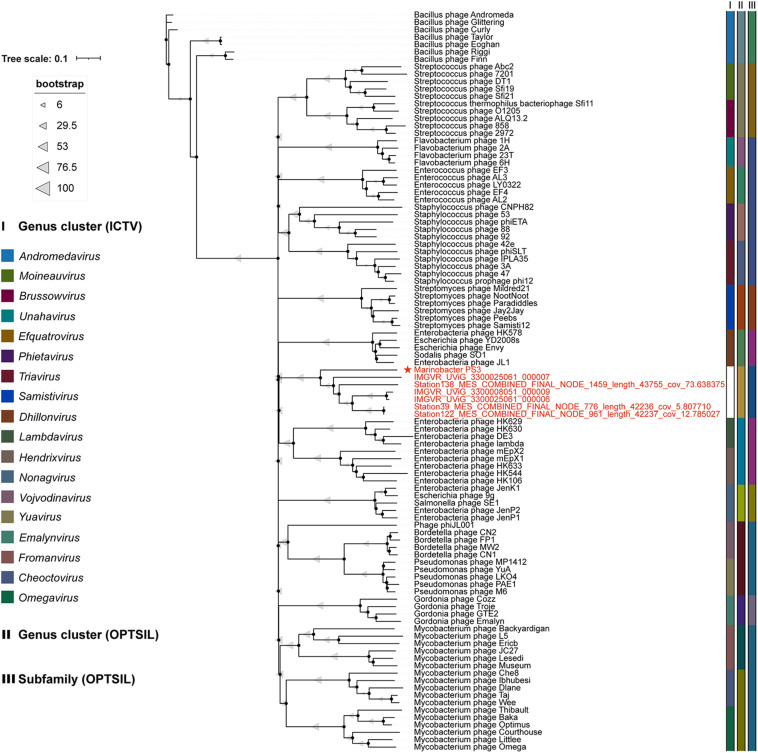
Genome BLAST distance phylogeny (GBDP) tree of 100 siphovirus genomes. vB_MalS-PS3 shows the phylogenomic GBDP trees inferred using the formula D6 and yielding average support of 57%. The numbers above branches are GBDP pseudo-bootstrap support values from 100 replications. The branch lengths of the resulting Virus Classification and Tree Building Online Resource (VICTOR) trees are scaled in terms of the respective distance formula used. The ICTV taxonomy and predicted OPTSIL taxon are shown. Each genus is indicated by a unique color. The members in VC 364_0 (Figure 4A) are shown in red.

Phages are regarded as members of the same genus by the International Committee on Taxonomy of Viruses (ICTV) when their nucleotide sequence identities are greater than 70% (Adriaenssens and Brister, 2017). The ANI value between vB_MalS-PS3 and the other six environmental viral sequences ranged from 69.97 to 100% (Figure 4B), confirming these phages (VC 364_0) might be identified as a new genus. But the application of ANI to taxonomy is limited to sequences with high coverage at the whole genome level (Feng et al., 2021). Therefore, whole-genome-based phylogenic analysis was conducted for vB_MalS-PS3 and the other six MAGs through VICTOR (Figure 5). The whole-genome phylogeny and OPTSIL taxon results are implied that vB_MalS-PS3 and the other six environmental viral sequences are clustering together (shown in red), comparing with the identified siphoviral genera in ICTV. In conclusion, all results confirmed that vB_MalS-PS3 and related MAGs are a novel unassigned viral genus, while vB_MalS-PS3 is the only isolates in this putative genus and named here as *Marinovirus.*

## Conclusion

Bacteriophages play a significant role in marine biogeochemical cycles by affecting their host’s physiology and ecology. In this study, we describe a novel Lambda-like temperate phage vB_MalS-PS3, which infects *M. algicola* DG893(T). In addition to the comparatively special position in phylogeny, the genomic signature of vB_MalS-PS3 shows a mosaic pattern as a novel temperate phage, which might make it a viral isolate, representing a genus of uncultured viruses, namely *Marinovirus*. Investigation of phage functional genes and other less well-studied genes will no doubt enable phage genome analysis to be more explicit and detailed. In subsequent studies on the phages-host system, some functional genes will need to be analyzed in more detail, including putative integration function, structural and morphogenesis genes, tape measure protein, other structural proteins, early regulation region, host lysis, DNA modification, small ORFs and other noteworthy features. Its evolutionary linage is novel and represents a cluster together with some uncultured virus sequences.

The study will contribute to the understanding of the role of lysogenetic viruses in marine ecosystems, particularly Marinobacter bacteriophages, including their host specificity, adaptation to host defense systems and propagation dynamics in natural systems. In the future, isolation, genome sequencing and interaction analysis of phages-hosts systems of *Marinobacter* can improve our understanding about their host defense systems and co-evolution relationships. Currently, databases of genes and proteins are extremely limited, and more phages need to be isolated and identified from different environments. Marinobacter phages have important effects, not only on lysing the host but also on the manipulating host to enroll the global biogeochemical circle through viral chronic infections. This study will contribute to the understanding of the physiological, genetic diversity and genomic characteristics of phages and their hosts in different aquatic environments. This will enable the exploration of the ecological characteristics of the system of phages-host under different conditions and the characteristics of their interactions.

## Data Availability Statement

The datasets presented in this study can be found in online repositories. The names of the repository/repositories and accession number(s) can be found in the article/Supplementary Material.

## Author Contributions

YTL and MW: conceptualization and project administration. YDL: methodology, writing, and original draft preparation. KZ: software. WZ, XZ, and HS: validation. YJ and YQJ: formal analysis. CG: investigation. HW: resources. SY: data curation. AM and BL: writing, review, and editing. YS, WM, and LW: visualization. HH: supervision. YTL, MW, and AM: funding acquisition. All authors contributed to the article and approved the submitted version.

## Conflict of Interest

The authors declare that the research was conducted in the absence of any commercial or financial relationships that could be construed as a potential conflict of interest.

## Publisher’s Note

All claims expressed in this article are solely those of the authors and do not necessarily represent those of their affiliated organizations, or those of the publisher, the editors and the reviewers. Any product that may be evaluated in this article, or claim that may be made by its manufacturer, is not guaranteed or endorsed by the publisher.

## Footnotes

1
http://ccb.jhu.edu/software/glimmer/index.shtml

2
http://rast.nmpdr.org

3
http://topaz.gatech.edu/GeneMark/

4
https://www.ncbi.nlm.nih.gov

5
https://pfam.xfam.org/search/sequence

6
http://lowelab.ucsc.edu/tRNAscan-SE/

7
https://www.ebi.ac.uk/interpro/

8
https://www.ncbi.nlm.nih.gov/cdd

9
https://www.genome.jp/viptree/

10
https://ggdc.dsmz.de/victor.php

11
http://jspecies.ribohost.com/jspeciesws/#analyse
